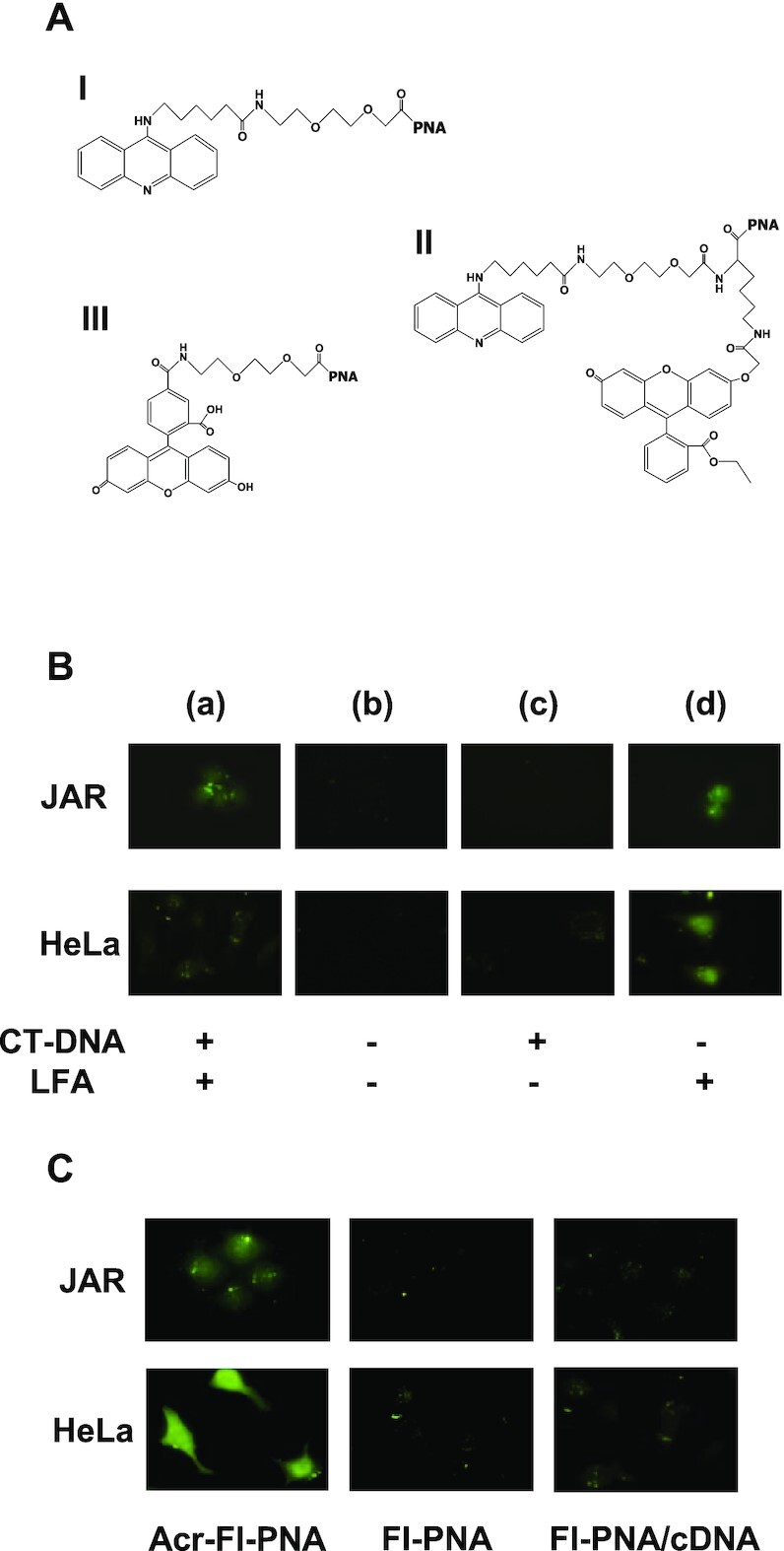# Editor's note on ‘down-regulation of MDM2 and activation of p53 in human cancer cells by antisense 9-aminoacridine–PNA (peptide nucleic acid) conjugates’

**DOI:** 10.1093/nar/gkad166

**Published:** 2023-03-02

*Nucleic Acids Research*, Volume 32, Issue 16, 15 August 2004, Pages 4893–4902, https://doi.org/10.1093/nar/gkh820

The Editors were alerted in November 2022 that the JAR (c) and HeLa pLuc705 (c) panels depicted in Figure 5B show unusual levels of similarity.

The experiments were conducted 20 years ago, and the authors no longer have the original data. The authors have retrieved and examined the submitted graphic files and agree that the two panels do indeed look very similar. The authors have provided an earlier figure, shown below, that was generated at a similar time from a replicate experiment. This figure confirms the results, namely that no significant fluorescence signal (cellular uptake) is observed for either cell type without Lipofectamine (LFA).

The authors conclude that the same image must have inadvertently been inserted twice at revision.

This error does not affect the results or conclusion of the article.